# Emerging insights into the impacts of heavy metals exposure on health, reproductive and productive performance of livestock

**DOI:** 10.3389/fphar.2024.1375137

**Published:** 2024-03-19

**Authors:** Ali Afzal, Naima Mahreen

**Affiliations:** ^1^ Animal Sciences Division, Nuclear Institute for Agriculture and Biology College (NIAB-C), Pakistan Institute of Engineering and Applied Sciences (PIEAS), Faisalabad, Pakistan; ^2^ School of Zoology, Minhaj University Lahore, Lahore, Pakistan; ^3^ National Institute for Biotechnology and Genetics Engineering College (NIBGE-C), Pakistan Institute of Engineering and Applied Sciences (PIEAS), Faisalabad, Pakistan

**Keywords:** heavy metals, toxicity, health, production performance, livestock

## Abstract

Heavy metals, common environmental pollutants with widespread distribution hazards and several health problems linked to them are distinguished from other toxic compounds by their bioaccumulation in living organisms. They pollute the food chain and threaten the health of animals. Biologically, heavy metals exhibit both beneficial and harmful effects. Certain essential heavy metals such as Co, Mn, Se, Zn, and Mg play crucial roles in vital physiological processes in trace amounts, while others like As, Pb, Hg, Cd, and Cu are widely recognized for their toxic properties. Regardless of their physiological functions, an excess intake of all heavy metals beyond the tolerance limit can lead to toxicity. Animals face exposure to heavy metals through contaminated feed and water, primarily as a result of anthropogenic environmental pollution. After ingestion heavy metals persist in the body for an extended duration and the nature of exposure dictates whether they induce acute or chronic, clinical or subclinical, or subtle toxicities. The toxic effects of metals lead to disruption of cellular homeostasis through the generation of free radicals that develop oxidative stress. In cases of acute heavy metal poisoning, characteristic clinical symptoms may arise, potentially culminating in the death of animals with corresponding necropsy findings. Chronic toxicities manifest as a decline in overall body condition scoring and a decrease in the production potential of animals. Elevated heavy metal levels in consumable animal products raise public health concerns. Timely diagnosis, targeted antidotes, and management strategies can significantly mitigate heavy metal impact on livestock health, productivity, and reproductive performance.

## Introduction

Technological developments have raised serious concerns about environmental safety as uncontrolled industrialization without emission control has put livestock health at risk (Prata et al., 2021). The livestock sector, an indispensable part of the global economy, is facing challenges because the production potential of animals depends on their health status, influenced by infectious and non-infectious diseases (Bailey et al., 2021). Heavy metal poisoning is one of the major causes of non-infectious diseases that adversely affect animal health. The risk of vulnerability is increasing due to widespread industrial, agricultural, domestic, technological, and medical applications of heavy metals (Vardhan et al., 2019).

Heavy metals like Zn and Cu are incorporated in animal feed as they play pivotal roles in immune function, growth, and metabolism (Hill and Shannon, 2019). These metals also serve as cofactors for numerous enzymes involved in protein synthesis and energy metabolism, thus promoting efficient nutrient utilization and growth in animals (Duffy et al., 2023). Moreover, Fe and Mn are indispensable for the formation of hemoglobin and enzymes involved in oxygen transport and antioxidant defense mechanisms (Pajarillo et al., 2021; Swecker, 2023). Adequate levels of these metals in livestock diets ensure optimal oxygen supply to tissues, which is particularly crucial for high-producing animals like dairy cows.

The escalating human activities contribute to a drastic increase in heavy metal addition to the environment, posing a continuous threat to livestock through water and soil pollution (Maurya et al., 2019; Ghazzal et al., 2022). The persistent exposure to these contaminants results in adverse health effects in livestock (Boudebbouz et al., 2023; Akbar et al., 2024). Additionally, the transfer of heavy metals from animals to humans through the food chain raises concerns about food safety and public health (Aftab et al., 2023; Noor et al., 2024).

The issue of metal toxicity has become a growing concern from evolutionary, ecological, environmental, and nutritional aspects particularly in the less developed countries of Africa, South Asia, and parts of Latin America (Pandey et al., 2014; Diarra and Prasad, 2021; Samy et al., 2022). In these countries, arid and semi-arid regions face particular challenges with metal pollution due to factors such as limited water resources, which concentrate pollutants, and the potential for windblown dust to spread contaminants over large areas (Chowdhury et al., 2016; Samy et al., 2022). Some countries in transition or with rapidly growing industrial sectors also face the challenge of heavy metal pollution due to lack of environmental regulations and limited resources for pollution control (Ding, 2019; Dutta et al., 2022; Patwa et al., 2022). While highly developed countries like the United States, China, Russia, and certain European nations have experienced issues with heavy metal contamination in soil, water, and air due to historical industrial practices (Li et al., 2014; Zhou et al., 2020). Altitude itself is not a direct factor in heavy metal pollution. However, certain activities more common at higher altitudes, such as mining in mountainous regions, can lead to localized heavy metal contamination. Additionally, factors like precipitation patterns and soil characteristics influenced by altitude can affect the transport and fate of metals in the environment (Giri et al., 2020).

The pervasive and mutinous nature of metal contaminants poses a potential risk for different animal populations. The distinct symptoms of metal poisoning in livestock include central nervous system (CNS) disorders, liver and kidney problems, reproductive failure, endocrine abnormalities, depression, and vision disturbances (Massányi et al., 2020; Rehman et al., 2021). If not diagnosed and treated properly, heavy metal exposure leads to complex problems with high morbidity and mortality rates. The sudden exposure to metals cause acute toxicity, with the severity of health issues depending on individual susceptibility, exposure route and duration, and the type and form of the element (Khan et al., 2016; Sinha et al., 2022). The pathophysiology of metals involves oxidative stress characterized by the generation of Reactive Oxygen and Reactive Nitrogen Species (ROS and RNS), reduction in intracellular free-radical scavengers and antioxidant stores, and decrease in enzyme capacity for detoxification of ROS. This leads to oxidative stress causing immunosuppression and a reduction in body condition scoring of domestic animals (Paithankar et al., 2021; Sun et al., 2022). Livestock populations are negatively influenced by exposure to excessive levels of harmful metals (e.g., Pb, Cd) or inadequate amounts of essential trace elements (e.g., Se and Mo) (Rajendran, 2013).

Ionic forms of metals exhibit high reactivity, engaging with biological systems in diverse ways that result in significant toxicological implications (Brandelli, 2020; Patel et al., 2021). The primary livestock species affected by metal poisoning in this scenario is cattle, mainly nourished with indigenously cultivated feed (Wang et al., 2013; Sharifian et al., 2023). To evaluate the potential effects of pollutants on livestock and measure contaminant intake in humans through milk and meat, it is essential to understand the concentrations of harmful metals in feed (Tortajada and Zhang, 2021; Ullah et al., 2023).

Research on the impacts of heavy metals exposure on the health, reproductive, and productive performance of livestock has garnered significant attention in recent years. Several published reviews have explored the diverse effects of heavy metals such as Pb, Cd, Hg, and As on various aspects of livestock physiology and productivity (Martinez et al., 2020; Sun et al., 2022; Duffy et al., 2023; Sadeghian et al., 2024). These reviews have elucidated the mechanisms by which heavy metals enter the animal body, their accumulation in tissues, and the resultant adverse effects on health. Some studies have highlighted the detrimental effects of heavy metals on immune function, oxidative stress, and reproductive functions in different livestock species (Garcia et al., 2017; Wang et al., 2019; Patel et al., 2021; Tahir and Alkheraije, 2023). Heavy metal contamination in livestock environments poses not only health risks to animals but also potential hazards to human consumers through the food chain. As such, this topic holds immense importance for informed decision-making in agricultural and veterinary practices. The study will comprehensively provide emerging insights and recent research findings regarding the effects of heavy metals on livestock. By collating and analyzing the latest scientific literature, this review aims to provide an overview of the current understanding of heavy metal toxicity in livestock and its implications for animal health and productive performance. Additionally, the review will explore potential mitigation strategies and areas for future research, contributing to the ongoing discourse on sustainable livestock management in the face of environmental challenges.

### Heavy metals and their biological significance

Metals are classified broadly into highly toxic, less toxic, essential, and non-essential categories. Essential metals like Fe, Zn, Mn, and Co are required in trace amounts for regulating various physiological functions such as hormone synthesis, oxygen and electron transportation, fertility, antioxidant defense, and immunity in livestock animals (Li et al., 2021). However, their presence beyond a certain limit in the biological system potentiates toxicity.

Highly toxic metals like As, Cu, Hg, Cd, and Pb can exert adverse effects even in minute concentrations without providing any beneficial biological impacts in animals (Li et al., 2014). Toxic metals impair the primary cellular functions by imitating the role of essential metals and this leads to their bioaccumulation. The elemental nature of metals affects the process of their biotransformation and detoxification through metabolic pathways that result in the breakdown of toxic atomic species into a less toxic or non-toxic species. In addition, the non-biodegradable nature of these metals further exacerbates the condition of toxicity (Florea et al., 2005).

### Sources of heavy metals contamination

**FIGURE 1:**
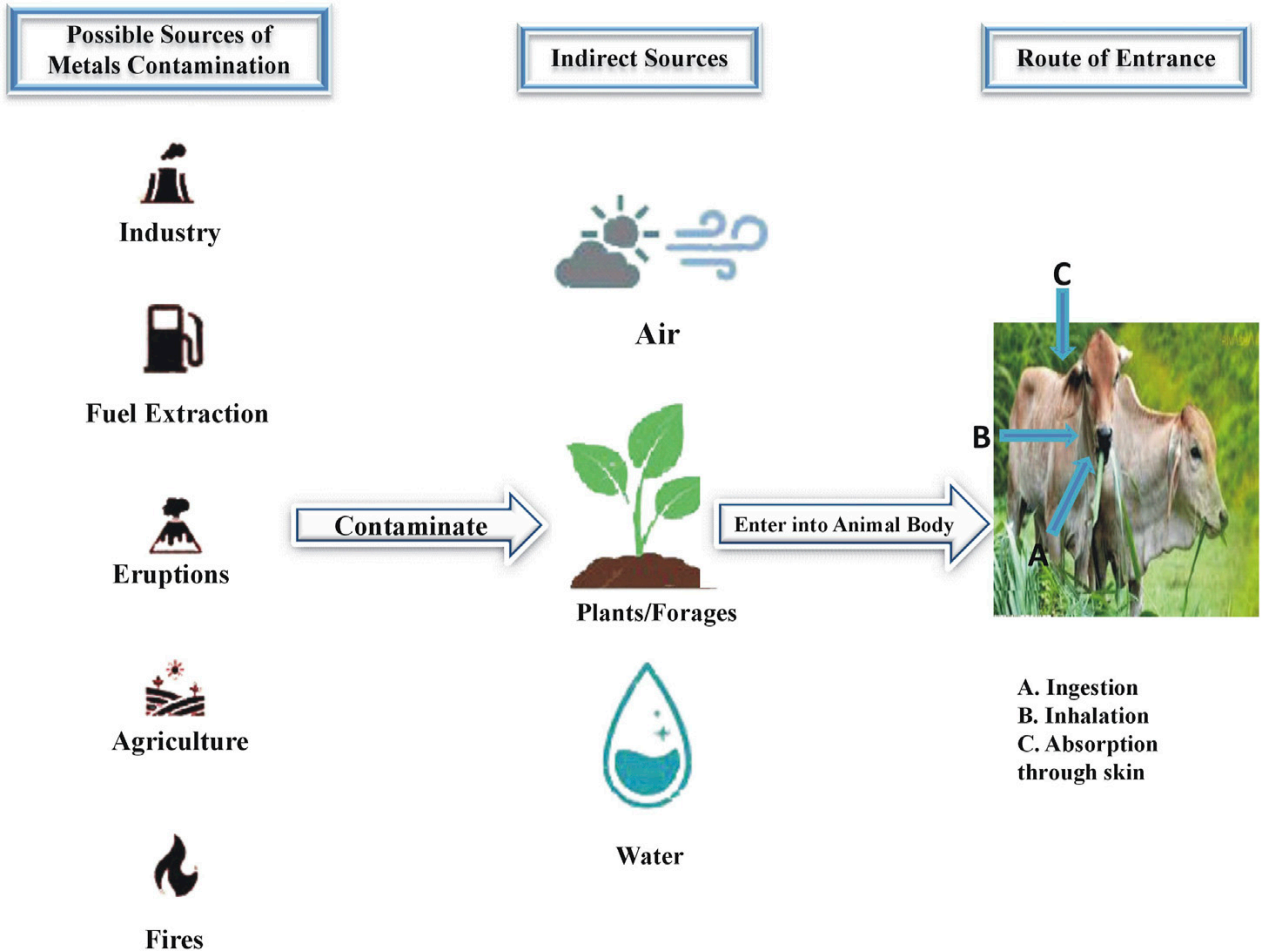
Sources of heavy metals contamination and routes of their entrance into animal body.

Sources of heavy metals contamination vary widely for livestock animals (Figure 1), with industrial effluents emerging as a significant contributor (Elheddad et al., 2021). Wastewater from industries introduces hazardous pollutants like Cr into the soil and water bodies (Dotaniya et al., 2014). This contamination poses threats to livestock as they may drink poisonous water or graze on polluted pastures. Similarly, automobile emissions contribute to metal contamination along roadsides. Livestock grazing near roads exposed them to metal pollutants that adversely affect their health (Ghorani-Azam et al., 2016).

Moreover, the application of fertilizers including phosphate fertilizers and organic materials (sewage sludge and animal manure) introduces heavy metals into agricultural soils (Rizzardini and Goi, 2014). Livestock may consume crops grown in such contaminated soils, resulting in the bioaccumulation of heavy metals in their tissues. Additionally, mining activities release toxic metals (As, Hg, and Pb) into the environment (Okereafor et al., 2020). Animals in mining areas may face direct exposure to these pollutants through soil, water, and vegetation, impacting their health, productivity, and welfare.

### Toxic metal contents in livestock feedstuffs

Livestock exposure to toxic metals is significantly influenced by husbandry practices. Sometime different dietary supplements and ingestion of contaminated soil also contribute to potential exposure to heavy metals. While evaluating the concentration of heavy metals in primary feed ingredients, it is crucial to consider husbandry practices as contributing factors to metal exposure. This consideration is essential to limit the toxic effects on animal health and the potential transfer of these contaminants to human feed. Providing a comprehensive review of toxic metal contents in animal feed proves challenging due to restricted information regarding sample description, analysis methods, and data expression. The limit of toxic heavy metals in both individual feed ingredients and commercially manufactured compound feeds is presented in Table 1 based on European Union (EU) organization standards (Adamse et al., 2017). Limited literature outside the EU provides information on toxic metal residues in livestock feedstuffs. Such data is often derived from samples collected at a very local level.

**TABLE 1:** Amount (mg/kg DM) of heavy metals in animal feed components.

Feed components	Heavy metals			
	Cadmium	Lead	Arsenic	Mercury
Barley Grain	0.11	0.97	0.01	0.006
Citrus Pulp Meal	0.19	0.76	0.04	0.002
Fish Meal	0.4	0.52	4.7	0.1
Maize Grain	0.06	0.56	0.26	0.026
Rapeseed	0.15	0.6	0.08	0.031
Soya Bean Meal	0.07	0.93	0.022	0.055
Sugar Beet Pulp	0.14	1.47	0.36	0.028
Sun Flower Meal	0.41	0.37	0.001	0.003
Mineral Supplements	0.58	3.38	6.8	0.02
Wheat	0.19	0.26	0.043	0.003
Meat and Bone Meal	0.063	0.81	0.005	0.15
Fish Oil	0.021	0.14	7.6	0.03
Oil Seed Meals	0.005	0.06	0.09	0.007
Cereal and By Products	0.03	0.048	0.06	0.011
Amount (mg/kg DM) of Heavy Metals in Animal Forages				
Forages	Heavy Metals			
	Cadmium	Lead	Arsenic	Mercury
Grass/Herbage	0.62	4.93	0.12	0.071
Hey	0.73	3.89	0.05	0.18
Grass Silage	0.09	2.02	0.12	0.023
Maize Silage	0.28	2.19	0.05	0.007
Different Straws	0.15	0.033	0.05	0.026
Alfalfa	0.057	0.21	0.38	0.005
Amount (mg/kg DM) of Heavy Metals in Complete Animal Feeds				
Animals	Heavy Metals			
	Cadmium	Lead	Arsenic	Mercury
Ruminants	0.11	0.34	0.27	0.012
Poultry	0.16	0.87	1.83	0.039
Pigs	0.09	1.03	0.62	0.032
Horses	0.21	0.13	0.39	0.022
Mink	0.003	0.28	0.061	0.053
Rabbits	0.14	0.29	0.09	0.031
Rodents	0.36	0.002	0.73	0.05
Dogs and cats	0.13	0.04	0.002	0.02

### Livestock husbandry practices related to toxic metal exposure

Livestock husbandry practices, particularly those involving feed supplements and their waste management; contribute to elevated levels of toxic metal exposure in animals, posing risks to their health and production (Wang et al., 2013). Feed supplementation is a primary pathway for toxic metal introduction in animal production systems. Heavy metals such as Cu and Zn are often added to animal feed to promote growth and boost immune function (Harvey et al., 2021). These metals are essential in trace amounts, over-supplementation or improper formulation of feed can lead to accumulation in animal tissues, potentially reaching levels harmful for the animals themselves and as well as consumers of their products.

Furthermore, the disposal of livestock waste presents another avenue for toxic metal contamination. Manure from animals fed supplemented diets can contain elevated levels of heavy metals, which may leach into soil and water systems if not managed properly. As a result, crops grown in these environments can uptake these metals, perpetuating the cycle of contamination (Liu et al., 2020a). In addition, the spreading of manure on agricultural fields as fertilizer can introduce heavy metals into the food chain, posing risks to human health through consumption of contaminated crops or water sources (Qin et al., 2021). The exposure of animals to toxic heavy metals during husbandry practices also depends on the type of production system (extensive or intensive) under which they are housed. Therefore, effective waste management strategies are crucial to mitigate the spread of toxic metals from livestock operations to the broader environment.

### Extensive production system

Regions of the world where animals are kept under extensive production systems, such as parts of South America, Africa, and Asia, are particularly vulnerable to heavy metal contamination due to factors such as industrial activities, mining operations, and improper waste disposal practices (Modernel et al., 2019; Otte et al., 2019; McAllister et al., 2020). The impact of heavy metals on livestock health and performance in these regions is most pronounced during the dry or winter seasons when forage availability is limited, and animals are forced to graze on contaminated vegetation or drink from contaminated water sources. During these periods, livestock animals experience heightened levels of heavy metal exposure, exacerbating the negative effects on their efficiency in extensive production system (Adegoke and Abioye, 2016; Johnsen and Aaneby, 2019). Prolonged exposure to elevated levels of these metals has led to various health issues, including gastrointestinal disturbances, liver and kidney damage, and neurological disorders. The presence immune system of animals is also compromised, reducing their resistance to pathogens and making them susceptible to infectious diseases (Villalba et al., 2016; Chen et al., 2022).

The heavy metal poisoning also disrupts the productive performance of animals in extensive system due to poor nutrient absorption and utilization. This leads to reduced feed efficiency, slower growth rate and decreased milk and meat production (Miroshnikov et al., 2021). Moreover, metal toxicity also causes metabolic disorders that further compromise the profitability of extensive livestock operations. In severe cases, metal poisoning results in acute illness and death, posing significant welfare concerns for animals (Okereafor et al., 2020). Effective management strategies, including regular monitoring of soil and water quality, implementation of proper waste management practices, and targeted nutritional interventions, are essential to mitigate the risks associated with heavy metal contamination and ensure the sustainability of extensive livestock farming operations (Qin et al., 2021).

### Intensive production system

Non-ruminant (pigs and poultry) animal species whose diet entirely consists of concentrates and ruminant animals (feedlots and milk production cattle) with a high rate (90%) of concentrates in their feed are managed under intensive production system (Moorby and Fraser, 2021). The feed for animals in this system is composed of various materials selected from national and international markets based on cost, availability, and nutritional suitability according to the physiological requirements of the animals.

The complete feed given to these animals is supplemented with specific feed additives and minerals that contain higher toxic metal contents compared to their basic feed. Due to low inclusion rate, the contribution of these supplements to total metal intake (TMI) is minimal, except for certain elements like Hg and As in marine-originated feedstuffs (Elliott et al., 2017).

In the intensive production system, toxic metal exposure is easily calculable by multiplying the toxic metal contents in the complete feed by the amount ingested. Furthermore, the diets in this system are highly standardized resulting in lower intra- and inter-farm variability of toxic metal exposure through the diet compared to the extensive system (Wang et al., 2013).

### Health risks and toxic effects of heavy metals in animals

Health risks and toxic effects of heavy metals in animals depend on the type and form of the metal, the extent of exposure, sex, age, nutritional and physiological status of the exposed animal, and the route of poisoning. Due to their non-biodegradable nature heavy metals tend to accumulate in vital organs more rapidly than they are metabolized and excreted. This bioaccumulation of heavy metals poses a significant health risk adversely affecting the production performance of domestic animals. The potential health menaces caused by various heavy metals are described below.

### Arsenic

Arsenic (As) known as the “Poison of Kings and the King of Poisons,” is an ancient toxin. Domestic animals face As exposure from contaminated pastures, dipping and spraying chemicals, pesticides, herbicides, growth promoters, and feed additives. Groundwater and metal-bearing ores are natural sources of As (Constable et al., 2017). After absorption As accumulates in the liver then slowly dispenses to the tissues of other organs like kidneys, spleen, and lungs. Animals in As toxicity exhibit gastrointestinal, nervous, and cardiovascular symptoms. The acute poisoning cases show restlessness, abdominal pain, respiratory distress, and vomiting (Gupta et al., 2021).

A meta-analysis of 156 cases in 16 outbreaks revealed common clinical signs in cattle including ataxia, diarrhea, dehydration, respiratory stress, and death (Faires, 2004). Hematuria, azotemia, increased hematocrit and liver enzyme activities were observed as biochemical changes (Bertin et al., 2013). Survival in acute As toxicity is possible with aggressive fluid therapy and antidote administration. Chronic As toxicity manifests as capricious appetite, weight loss, mucosal lesions, mouth ulceration, and reduced milk yield in dairy cattle. Biochemical changes in subclinical cases include high blood as levels, low plasma proteins and erythrocyte count (Rana et al., 2010).

Quantification of As in stomach contents and tissues is a standard test for diagnosis. While for sample collection liver is regarded as the best organ for acute poisoning and kidneys for sub-acute or chronic cases (Constable et al., 2017). Levels exceeding 3 ppm in liver and kidneys indicate toxicity. As also appears in urine samples a few days after exposure (Sharaf et al., 2020). The diagnostic range for As in feces is 10–20 mg/g, while As quantification in hair and urine serves as a biomarker for monitoring chronic exposure in livestock (Das et al., 2021; Tiffarent et al., 2022).

### Lead

Lead (Pb) recognized as the first toxic element that poses a higher frequency of poisoning in animals than any other metal (Gupta, 2019). This issue spans worldwide affecting all species of livestock, with cattle and calves being susceptible while caprine and swine show tolerance. Cattle due to their indiscriminate eating and licking habits ingest Pb-bearing objects from their environment including agricultural, industrial, and domestic wastes resulting in acute Pb poisoning (Liu et al., 2020b). Apart from natural exposure anthropogenic activities also introduce Pb into the food chain through contaminated feed and water, burning of fossil fuels, paint industries, and combustion of coal. (Zyambo et al., 2022). Pastures near highways may carry up to 390 ppm Pb (Anil et al., 2021). Organic Pb compounds are more toxic than inorganic forms and animals with essential dietary mineral deficiencies are more prone to lead toxicity (Prabhakar et al., 2012; Su et al., 2022).

Pb has ability to cross blood-brain and placental barriers, so it adversely affects the brain and fetus of exposed animals. Clinical signs manifest due to chronic exposure when Pb levels surpass saturation limits in natural sinks like bones (Constable et al., 2017). Pb affects various physiological functions leading to gastrointestinal damage, neurotoxicity, liver and kidney dysfunctions, oxidative stress, and disruptions in enzyme systems (Finnie et al., 2011; Patra et al., 2011; Slivinska et al., 2020). Clinical signs in Pb poisoning mainly result from toxic effects on the CNS, gastrointestinal tract (GIT), and haemopoietic system. Neurological signs include bellowing, blindness, dullness, head pressing, opisthotonus, convulsions, and coma. Gastrointestinal signs include distension, pain, cramping, and diarrhea. Sub-acute Pb toxicity presents anorexia, dullness, depression, weight loss, eye sight loss, staggering, in co-ordinations, and sometimes circling, followed by death (Krametter-Froetscher et al., 2007; Mukherjee et al., 2022). Pb also affects the reproductive system impacting animal productivity (Okereafor et al., 2020). Pb exposure alters essential trace mineral profiles, with cows exhibiting lowered blood Cu and Fe at blood Pb levels >0.60 μg/mL (Patra et al., 2006). Some studies show higher tolerance to Pb in sheep and goats than cattle as these animals excrete higher concentration of Pb in their excretions (Makridis et al., 2012). Goats being highly resistant may show signs of CNS involvement following long-term exposure (Swarup et al., 2005).

Diagnosis is based on significantly high Pb concentrations in blood, liver, and kidney samples (Oluokun et al., 2007). Management and prevention involve identifying sources and adopting preventive measures providing supportive care with chelation therapy and ensuring food safety. Calcium versenate is an effective antidote with combination therapy showing promising results. Recovery may occur rapidly in less severe cases but Pb concentrations in tissues remain high posing a potential source of residues in the food chain (Siddiqui and Rajurkar, 2008).

### Mercury

Mercury (Hg) exists naturally in elemental, organic, and inorganic forms in the environment. Historically, it was used in traditional Chinese and Indian medicines. Different sources of Hg including vinyl chloride, chlor-alkali, electrical and electronic batteries, posing a potential threat to marine ecosystems through industrial effluents (Barregard et al., 2022). Domestic animals are less prone to Hg poisoning. However, accidental cases may occur especially in cattle (de Los Santos et al., 2023). Hg exposure stems from ingestion of organic or inorganic compounds, while toxicity observed at an average daily intake of 10 mg per kg body weight. Sheep and horses also exhibit toxic effects under certain intake levels (Constable et al., 2017). Hg absorption occurs through the GIT and then it affects other vital organs of the body (Bubber, 2019). Hg is slowly excreted through feces, milk, and urine, making the liver, kidneys, and milk from poisoned animals unsafe for human consumption (Bubber, 2019; de Los Santos et al., 2023).

Some organic Hg compounds are neurotoxic, affecting the nervous system even at lower concentrations (Ceccatelli et al., 2010). Inorganic Hg adversely affects the GIT mucosa causing vomiting, diarrhea, and colic, with acute poisoning signs in cattle including sudden behavioral changes, blindness, convulsions, and death (Krametter-Froetscher et al., 2007). Diagnostic importance lies in Hg concentration in blood, urine, brain, kidney, liver, and hair. Kidneys are ideal for detecting Hg poisoning in deceased animals, with a tissue concentration of >5 ppm regarded as clinically toxic. Blood Hg concentration provides more information about acute exposure, while urine samples determine inorganic Hg poisoning. Combining blood, urine, and hair samples provides accurate information about different forms of Hg exposure (Gupta, 2018). Effective treatment involves using 2,3-dimercapto-propanesulphonate (DMPS) and Meso-2,3-dimercaptosuccinic acid (DMSA) which mobilize Hg deposits for excretion in urine. Se supplementation in the diet proves useful in protecting against organic and inorganic Hg toxicity in poultry, as Se and Hg are mutual antagonists. Phytoremediation, utilizing plant species like *Brassica juncea* has emerged as a proven approach to reduce Hg contamination in soil (Ekino et al., 2007). However, multidisciplinary research is essential to manage Hg toxicity and understand various treatment mechanisms.

### Cadmium

Cadmium (Cd) a ductile and soft element with toxic properties, poses severe damage to vital organ systems (Rahimzadeh et al., 2017). It enters the environment through natural (volcanic activities, mineral ores, forest fires) and anthropogenic (processing facilities, non-ferrous metal smelters) sources, often released in combination with metals like Pb and Zn (Robards and Worsfold, 1991; Suhani et al., 2021). It is usually found as an impurity along with Zn and Pb deposits and therefore can be extracted as a byproduct during the smelting process of these metals. Rock phosphate fertilizer and sewage sludge leakage contribute to soil contamination leading to Cd transfer to plants and the food chain (Rahimzadeh et al., 2017). Domestic animals are exposed to Cd mainly through ingestion of plants grown in contaminated soils or in the vicinity of industrial units emitting Cd in the environment. Cd is mainly accumulated in leaves that act as a potential source of exposure to grazing animals (Madejon et al., 2009). Studies in Croatia and China revealed Cd concentrations exceeding limits in kidneys and liver, indicating environmental contamination (Cai et al., 2009; Bilandzic et al., 2010). Water from deep wells in certain areas may also carry high Cd levels (Perez-Carrera et al., 2016).

Cd ingestion causes acute liver damage and nephrotoxicity, while chronic exposure leads to organ accumulation and various clinical signs including in-appetence, weight loss, hoof and hair abnormalities (Constable et al., 2017). Severe vascular degeneration and necrotic changes occur in vital organs around industrial areas (Gumasta et al., 2018). Experimental administration in sheep resulted in nephropathy, anemia, bone demineralization, congenital defects, stillbirths, and abortion (Constable et al., 2017).

Limited exposure to Cd leads to subclinical effects like immunotoxicity, oxidative stress, reduced reproductive performance, endocrine disruption, altered micronutrient profiles, and poor weight gain (Swarup and Dwivedi, 2002). Long-term exposure causes lipid peroxidation, inhibits antioxidant enzymes, indicating oxidative damage in liver, kidneys, and testes (Wang et al., 2020). Ca, Fe, and Zn deficiency increases susceptibility to Cd toxicity, while Se supplementation protects the liver and kidneys (Alonso et al., 2004; Jemai et al., 2020). Including Mo and Fe in the diet prevents Cd poisoning signs (Smith and White, 1997; Alonso et al., 2004; Young et al., 2019).

### Copper

Copper (Cu) is an essential element crucial for various enzyme systems in the body, but excessive intake can lead to severe toxicity in farm animals particularly in ruminants lacking efficient regulatory mechanisms for Cu (Trouillard et al., 2021). Sheep are highly susceptible to Cu poisoning while cattle show relative resistance, though cases are increasing especially in dairy cattle (Lopez-Alonso et al., 2017).

Ruminants have poor homeostatic mechanisms for Cu, storing excess in the liver. When exposed to levels beyond physiological requirements they fail to regulate it leading to severe Cu toxicity (National Research Council, 2005; Almeida et al., 2022). Acute poisoning in sheep and young calves occurs with an intake of 20–100 mg/kg of body weight (BW), while adult cattle require 200–800 mg/kg BW. Chronic poisoning in sheep results from a daily intake of 3.5 mg/kg BW from pastures with 15–20 ppm Cu on a dry matter basis (Gupta, 2018). Goats tolerate higher Cu dietary intake (640 mg/kg BW) and toxic doses for non-ruminants are higher than this (National Research Council, 2005; Constable et al., 2017). Cu absorption varies in animals influenced by diet, husbandry practices, and breed types (Lopez-Alonso and Miranda, 2020).

Acute Cu poisoning in animals exhibits signs like gastric pain, diarrhea, anorexia, excessive salivation, dehydration, collapse, and incoordination, with some survivors developing icterus and dysentery (Elliott et al., 2020). Chronic poisoning manifests as hemolytic conditions, causing thirst, anorexia, depression and jaundice along with nervous signs (Quevedo et al., 2022). Cattle and buffaloes in chronic Cu poisoning show apathy, hemoglobinuria, reduced ruminal movements, dehydration, mild diarrhea, and icteric mucus membrane. Cu toxicity affects reproductive performance causing disorders related to pregnancy and influencing estrogen receptors (Hinde, 2021).

Diagnosis involves assessing Cu concentrations in tissues with liver biopsy being the most authenticated method. Serum Cu and cerulo-plasmin levels in live animals are commonly used parameters. Toxic levels in liver and kidneys are >250 ppm and 10 ppm in cattle, and >250 ppm and >18 ppm in sheep (Lopez-Alonso et al., 2006; Dalefield, 2017). Treatment involves using Mo and chelating agents (Antonelli et al., 2016; Mecitoglu et al., 2017; Martins et al., 2020). Zn supplementation is effective in preventing excessive Cu accumulation in sheep (Minervino et al., 2009). The potential health risks of heavy metals along with their maximum dose limits are summarized in Table 2.

**TABLE 2:** Heavy metals and their associated health risks in animals.

Heavy metal	Form	Sources	Entry routes	Maximum daily dose (μg/day) limits		Associated symptoms	Pronounced health effects	References
				Parenteral	Oral/Topical/Mucosal			
Arsenic (As)	Inorganic	Pesticides, Contaminated water	Ingestion, Inhalation	1.5	15	Skin lesions, Gastrointestinal issues, Neurological disorders	Skin cancer, Diabetes Cardiovascular disease	Faires, (2004); Rana et al., (2010); Bertin et al., (2013); Gupta et al., (2021)
Lead (Pb)	Inorganic	Contaminated soil, Water, Air	Ingestion, Inhalation	1	10	Anemia, Neurological disorders, Developmental delays	Impaired cognitive functions, Reproductive issues	Krametter-Froetscher et al., (2007); Finnie et al., (2011); Patra et al., (2011); Okereafor et al., (2020); Slivinska et al., (2020); Mukherjee et al., (2022)
Mercury (Hg)	Organic, Inorganic	Coal combustion, Seafood	Ingestion, Inhalation	1.5	15	Neurological disorders, Tremors, Developmental delays	Growth retardation, Impaired vision, Kidney damage	Krametter-Froetscher et al., (2007); Ceccatelli et al., (2010); Bubber, (2019)
Cadmium (Cd)	Inorganic	Industrial waste, Fertilizers	Ingestion, Inhalation	0.5	5	Renal dysfunction, Osteoporosis, Gastrointestinal problems	Kidney damage, Bone demineralization, Cancer risk	Swarup and Dwivedi, (2002); Cai et al., (2009); Bilandzic et al., (2010); Gumasta et al., (2018); Wang et al., (2020)
Copper (Cu)	Inorganic	Waste water, Feed supplements, Copper-based fungicides	Ingestion	250	2,500	Liver damage, Gastrointestinal disturbances	Hemolytic anemia, Cirrhosis, Neurological issues	Elliott et al., (2020); Hinde, (2021); Quevedo et al., (2022)

### Effects of heavy metals on reproductive performance

#### Male reproductive performance

**FIGURE 2:**
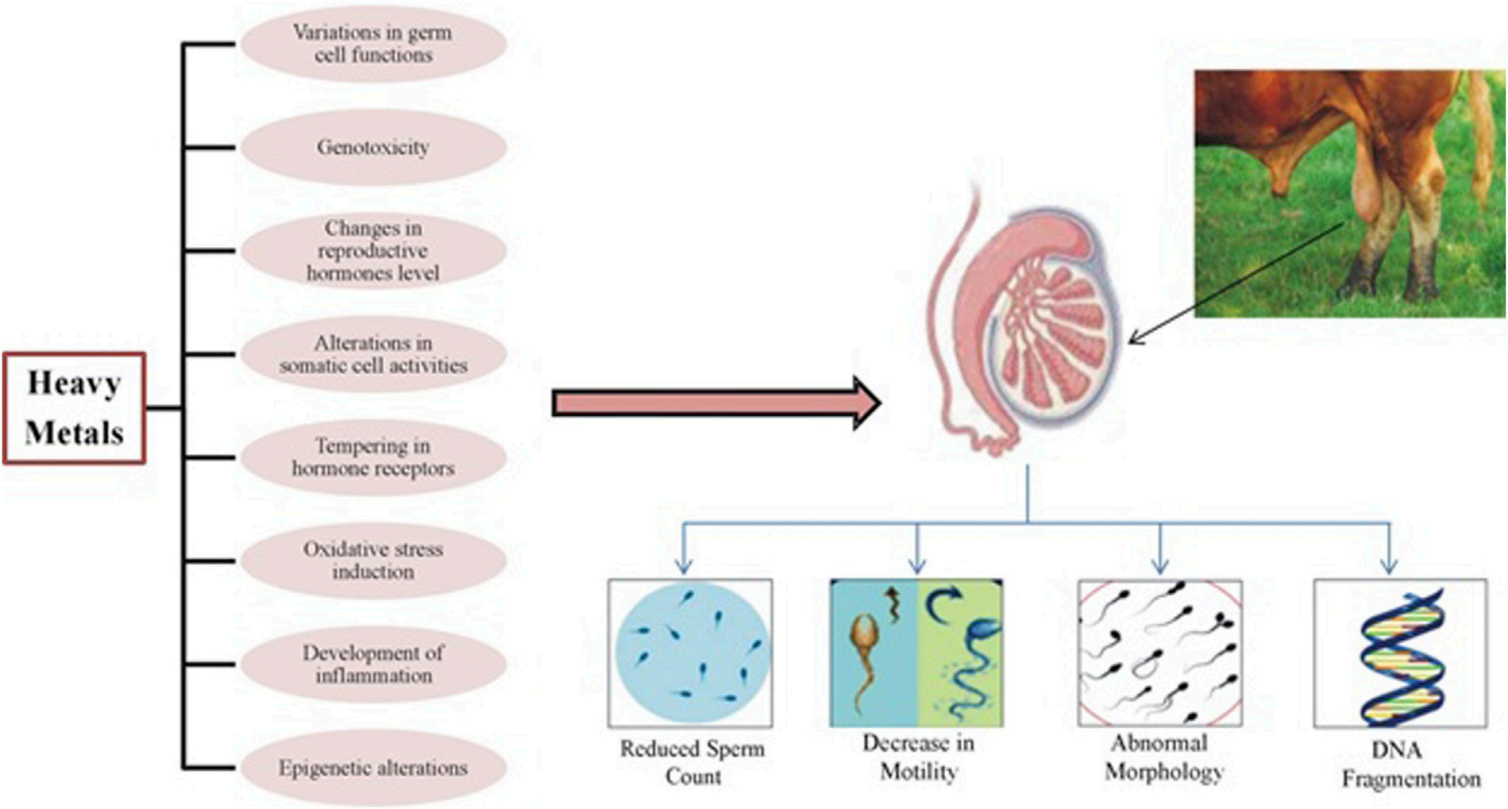
Adverse effects of heavy metals on sperm production.

Heavy metal toxicity has adverse effects on male reproductive performance impacting sperm maturation, concentration, and motility as shown in Figure 2 (Ilieva et al., 2020). In some livestock animal species sperm antioxidant defense system is poorly developed, so heavy metals disrupt normal motility and symmetry by accessing flagellum proteins through the sperm plasma-lemma (Kanous et al., 1993). Metal exposure in other species negatively affects the expression of metallo-thionein mRNA in testicular tissue crucial for protecting spermatogenesis from adverse effects of harmful substances (Sheng et al., 2015).

Mammalian testes are particularly susceptible to metal toxicities undergo remodeling of gene expression, alkalization of luminal fluid, and destabilization of sperm chromatin (Machado-Neves, 2022). Metals like Cd and Pb inhibit androgen production, microtubule movement, and the expression of regulatory protein genes resulting in a drastic decline in sperm count and histopathological changes in the testis (Bhardwaj et al., 2021; Salihu et al., 2021). Metal exposure also initiates testicular damage by altering angiogenesis processes (Rzymski et al., 2015). Cd exposure to Leydig cells increases cytokine production, inducing DNA breaks and reducing testosterone production leading to male infertility (Yang et al., 2022). Cd also stimulates α-adrenergic receptors producing excess glucocorticoids, inhibiting steroidogenesis and causing Sertoli cell damage (de Angelis et al., 2017; Slaby et al., 2019). A positive correlation between blood Cd levels and sperm abnormalities and an inverse relation with sperm density has been observed (Meligy et al., 2019).

Blood-Testicular Barrier (BTB) disruption due to heavy metal toxicity distorts tight junctions that negatively influences spermatogenesis and increases sperm abnormalities (Mukherjee et al., 2022). DNA hypermethylation due to metal toxicity leads to the overexpression of DNMT3b causing malignancy of prostate epithelial cells and permanent infertility (Anđelković et al., 2023). Further, metal toxicities may result in conditions like teratozoospermia, asthenozoospermia, reduced sperm surface mannose expression, loss of sperm head actin, acrosome reaction insufficiency, increased apoptosis, and altered transvascular fluid exchange with varicocele (Anđelković et al., 2023). Varicocele affects semen quantity and quality by influencing sperm count, morphology, and motility (Ilieva et al., 2020). Certain other biochemical and histological changes are also associated with heavy metals that adversely influence the reproductive performance of animals (Kar et al., 2015). Heavy metal in low concentration for a short period of time does not significantly affect reproductive performance of animals but moderate to high level in blood leads to poor semen quality and also reduces the reproductive hormones levels (Bhardwaj et al., 2021).

### Toxic effects on female reproductive performance

**FIGURE 3:**
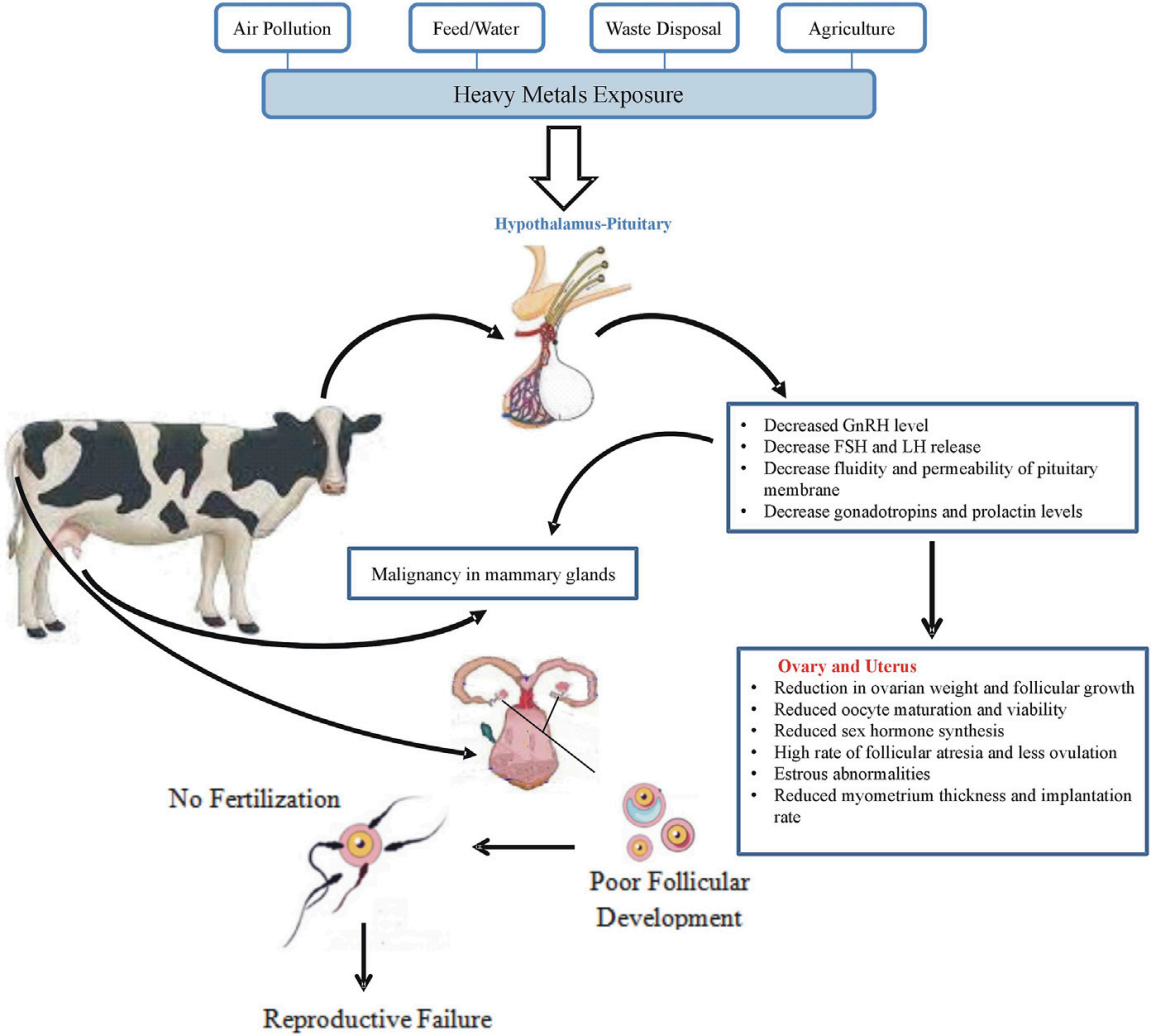
Reproductive failure in response to heavy metals exposure in cattle.

The exposure of female animals to heavy metals affects oocyte maturation rate, germinal vesicle breakdown, and sex organ weight causing impairment in the reproductive process (Figure 3) (Fort et al., 2001; Dutta et al., 2022). During the embryonic development process, 17β-estradiol was found to synergistically modify the harmful effects of heavy metals on the female reproductive system (Chedrese et al., 2006). Metals like Cd disrupt the functions of the reproductive endocrine system, impairing oocyte release from graafian follicles, affecting ovulation, and ultimately impacting animal breeding (Fort et al., 2001; Dutta et al., 2022).

DNA damage induced by heavy metals leads to excessive ROS production, causing oocyte death during meiosis-I and reducing the number of oocytes entering Metaphase-II stage (Engwa et al., 2019). Chronic metal exposure inhibits the functional activity of the p450 gene that influences the morphology and physiology of ovarian granulosa cells (Chedrese et al., 2006). Heavy metals interfere with FSH production, binding with its receptor and lower the rate of steroid synthesis in ovarian granulosa cells that affect steroidogenesis (Kirmizi et al., 2020).

Metal ions enter cells through L-type voltage-gated channels inhibiting Ca^2+^ ATPase pump activity and accelerating the expression of ERK, p38, and c-jun, crucial for granulosa cell proliferation and steroidogenesis (Sun et al., 2022). Some metals like Cd^2+^ replace Zn^2+^ in the regulatory domain of estrogen receptors (ERs) causing adverse changes in DNA binding domain elasticity and variations in steroidogenic gene expression at the transcriptional level (Paithankar et al., 2021). The development process of mammary glands is also adversely affected by the presence of metal ions inducing early arrival of puberty (Migdal‚ et al., 2023).

Changes in estrogen and progesterone receptors due to heavy metal alterations act as causative agents for reproductive issues like spontaneous abortion, endometrial cancer, and estrogen-dependent diseases of mammary glands (Bhat et al., 2023). Metals like Cd can replace bivalent ions (Ca^2+^ and Zn^2+^), and their deficiency during pregnancy increases the chances of spontaneous abortion. *In vitro* and *in vivo* studies show that Cd^2+^ exposure unfavorably influences oocyte maturation and causes chromosomal abnormalities (Leoni et al., 2002; Jia et al., 2011; Aglan et al., 2020).

The influx of metals in females increases during nutritionally critical stages (pregnancy and lactation), decreasing essential mineral elements (Zn^2+^, Fe^2+^, Cu^2+^) and promoting metal-induced reproductive toxicity. Metal exposure during pregnancy increases the chances of preterm labor, premature delivery, and low birth weight (Eaves and Fry, 2023). Tobacco smoke a source of metallo-toxins decreases female reproductive performance and causes structural changes in the fallopian tube and uterus (Semczuk and Semczuk-sikora, 2001).

Heavy metals can accumulate in the placenta of some animal species, affecting progesterone synthesis, inhibiting the transcription of essential enzymes, and preventing the maintenance of pregnancy (Thompson and Bannigan, 2008; Caserta et al., 2013; Geng and Wang, 2019). *In vitro* administration of different heavy metals in endothelial cells promotes the synthesis of placentation growth factor (PLGF) and vascular endothelial growth factor-A (VEGF-A) by mediating certain changes in mRNA expression. The transcription of the growth factors (VEGF-A and PLGF) alters the process of angiogenesis that is required for placentation, implantation and embryogenesis (Yüksel et al., 2021). The defects in expression of VFGF-A and PLGF lead to lack of proper implantation of embryo, endothelial dysfunction, sub-infertility, pre-eclampsia and premature delivery (Rzymski et al., 2015; Mishra and Bhardwaj, 2018).

### Toxic metals and animal products quality

Heavy metals present a significant concern in the quality of animal products reared under commercial conditions as they are more susceptible to contamination from environmental pollution and industrial wastes (Hejna et al., 2019). While organic farming of animals emphasizes natural methods of production, including pasture-based systems and restricted use of synthetic inputs such as pesticides and fertilizers, organic animals are less likely to be exposed to heavy metals from industrial sources such as contaminated soil, water, and feed additives. Among heavy metals contamination in commercial production of animals, As stands out as a particular concern as livestock animals can experience its toxic effects through the consumption of contaminated feedstuffs. As is primarily excreted in the milk of exposed animals, while its residues have also been detected in chicken, duck, cow, and goat meat (Zahrana and Hendy, 2015). As contamination deteriorates milk and meat quality that poses a serious risk to consumer health. Human exposure to As occurs through the consumption of contaminated foodstuff (milk and meat) as highlighted by a survey conducted by the European Food Safety Authority (EFSA) (Authority et al., 2021).

Despite the absence of a universally accepted safe limit for As in food, WHO has set an acceptable intake level of 3.0 g/kg BW for this toxic metal (Islam et al., 2014). The detection of As in cow’s milk and poultry liver suggests them as preferred deposition sites compared to other meat parts (Das et al., 2021). Notably, milk contains inorganic As due to the inability of methylated As to cross the udder epithelium of cows. The highest accumulation of As occurs in casein (83%), while fat, whey protein and skimmed milk contain minute amounts of 10%, 4%, and 3% respectively. This highlights the complexity of metal distribution within dairy products (Das et al., 2021).

Moreover, milk can also be affected to some extent by Cr and Ni (Somasundaram et al., 2005; Ubwa et al., 2017). Some other potential sources of contamination include milk utensils, feed, and the animals’ environment (Ismail et al., 2019). A study involving Jersey cows substantiated the bioaccumulation of toxic metals in milk with concentrations of Pb, Cd, Ni, and Cr reaching significant levels (Somasundaram et al., 2005). Interestingly, Pb concentrations exhibit an increasing pattern in the initial stages, followed by a stable level over time, contrasting with Cd levels that double after 10 days and remain constant thereafter (Somasundaram et al., 2005).

Feed additives and industrial emissions lead to varying heavy metal concentrations in the muscle tissue of pigs. A significantly high concentration of Pb (0.05–0.58 mg kg^−1^ d.m.) and Cd (0.02–0.04 mg kg^−1^) has been reported in the muscles of fattening pigs (Medardus et al., 2014; Chałabis-Mazurek et al., 2021). Sheep reared under pasture and extensive systems accumulate Cd in kidneys and udder, while Pb, Zn and Cu are predominant in ribs, liver, and long bones (Abd-Elghany et al., 2020).

Poultry, especially from backyard rearing systems accumulate heavy metals from sources like landfills and contaminated soil, resulting in exceeding permissible limits in meat and eggs. Duck and goose reared near industrial areas show higher concentrations of As, Cd, and Hg in their eggs as compared to non-industrial sites (Korish and Attia, 2020; Kabeer et al., 2021). Bees serve as bioindicators of toxic metal contamination and retain a significant portion of these metals from their surrounding environment during honey processing (Bosancic et al., 2020). Horse muscles generally lack excess heavy metals, but kidneys and liver often accumulate Cd and Zn (Gupta et al., 2021). Wildlife in industrially exploited regions exhibits levels of heavy metals surpassing legal limits for human consumption, with wild boar being the most affected species (Verma et al., 2023).

### Cytotoxic and oxidative effects of heavy metals

Pb exerts its cytotoxic effects by interfering with enzymes involved in heme synthesis, such as δ-aminolevulinic acid dehydratase (ALAD) leading to anemia (Ebrahimi et al., 2023). Further, Pb disrupts calcium signaling pathways by interfering with calcium-dependent processes that affects neurotransmitter release and synaptic plasticity (Cory-Slechta et al., 2016). Hg impairs mitochondrial function and energy metabolism, causing cellular dysfunction and apoptosis (Ke et al., 2023). Moreover, by disrupting neurotransmitter signaling, Hg interferes with the function of ion channels and neurotransmitter transporters, contributing to neurotoxicity (Novo et al., 2021). Cd binds to sulfhydryl groups in proteins. This binding inhibits their activity and disrupting cellular functions (Genchi et al., 2020; Suhani et al., 2021). As toxicity inactivates enzymes involved in cellular metabolism, including those in the tricarboxylic acid (TCA) cycle and oxidative phosphorylation (Machado-Neves and Spuza, 2023). As also disrupts cell cycle regulation, resulting in aberrant cell proliferation and apoptosis resistance (Naujokas et al., 2013).

Heavy metals induce the generation of highly reactive free radicals causing DNA damage, oxidation, protein depletion, lipid peroxidation, and various cytotoxic effects. The toxicity of different metals is primarily attributed to the production of ROS and RNS disrupting cellular redox potential. ROS with their high chemical reactivity produce free radicals like hydroxyl (OH), superoxide (O^2-^), alkoxyl (RO), and peroxyl (RO^2^) as well as non-radicals such as H_2_O_2_ and peroxynitrite (ONOO^−^) acting as oxidizing agents or converting into other radicals.

Intracellular, superoxide anion (O^2-^) generation involves redox components (e.g., semi-ubiquinone), enzymes (e.g., NADPH-oxidase (NOX), xanthine-oxidase), or auto-oxidation reactions (Brochin et al., 2008; Flora et al., 2012; Alkadi, 2020; Sylvester et al., 2022). Superoxide anion (O^2-^) exhibits limited reactivity under specific physiological conditions and cannot cross biological membranes. However, its reaction with nitric oxide (NO) leads to the production of peroxynitrite (ONOO^−^) with highly reactive intermediates like the short-lived hydroxyl radical (OH) (Pastor et al., 2000).

Involvement of nitric oxide synthase isozymes, such as mitochondrial nitric oxide synthase (mtNOS) and endothelial nitric oxide synthase (eNOS) also result in the production of nitric oxide (NO) from L-arginine to citrulline. NO due to its amphipathic nature remains stable in anaerobic environments and can diffuse through plasma membranes and cytoplasm. Its interaction with superoxide anion results in the production of peroxynitrite (ONOO^−^) (Szabo et al., 2007). An imbalance in ROS and RNS production or a decrease in scavenging activities as a consequence of external stimuli like metal exposure leads to alterations in cellular functions through direct changes in biomolecules and aberrant stimulation of specific signaling pathways that directly affect growth performance and production potential of animals.

### Epigenetic modifications

Epigenetic modifications such as DNA methylation, DNA repair, histone modifications, transcription, RNA regulation, RNA stability, protein degradation, transposon activation, and gene copy number alterations are intricate processes influenced by pollutants containing heavy metals. These metals induce gene alterations, contributing to complex changes responsible for malignancies, allergic reactions, respiratory diseases, intrauterine growth inhibition, pre-eclampsia, and permanent infertility in affected animals (Edwards and Myers, 2007; Godfrey et al., 2016).

**FIGURE 4:**
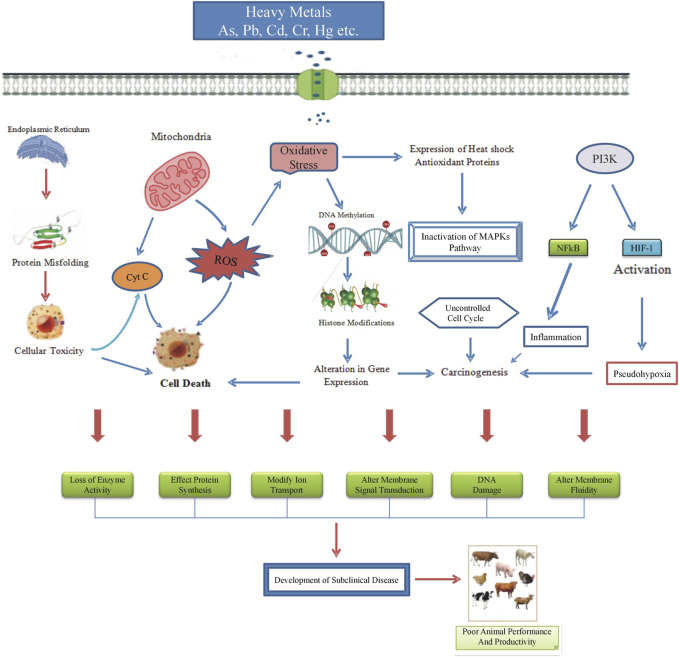
Mechanism of heavy metals induced cytotoxic and oxidative stress. Heavy metals negatively impact the process of protein biosynthesis, resulting in cellular toxicity. This disruption may induce the release of cytochrome c (Cyt c) from mitochondria, ultimately causing cellular apoptosis. Additionally, heavy metals contribute to the heightened generation of reactive oxygen species (ROS), leading to the induction of oxidative stress (OS). The consequences of OS include oxidative damage to cellular macromolecules, ultimately culminating in cell death. Furthermore, OS mediates epigenetic changes in reproductive cells, such as DNA methylation and histone modifications, altering the expression of crucial cellular proteins. The adverse effects extend to the inactivation of heat shock antioxidant proteins, disrupting the mitogen-activated protein kinases (MAPKs) pathways. This disruption results in uncontrolled cellular proliferation and may even contribute to carcinogenesis. Moreover, heavy metals activate the phosphoinositide 3-kinase (PI3K) pathway and induce hypoxia-inducible factor 1 (HIF1), promoting cancer, angiogenesis, and various cellular processes. The activation of the nuclear factor kappa-light-chain enhancer of activated B cells (NF-κB) by heavy metals also contributes to carcinogenesis. These cytotoxic and oxidative effects have a detrimental impact on the reproductive and productive efficiency of animals.

Current interest in epigenetics research and evidence of environmental variables influencing DNA methylation has expanded the study of health-related causes (Salnikow and Zhitkovich, 2008; Arita and Costa, 2009; Larson et al., 2010). Alteration in protein synthesis mechanisms due to heavy metal pollutants in the nucleus may lead to cellular toxicity, initiating the process of cell apoptosis (Figure 4) (Florea et al., 2005). In response to As, epigenetic modifications involve glutathione-reactive oxidation, increased expression of proteins (e.g., alpha-B-crystallin, heat-shock anti-oxidative stress proteins ferritin light chain) and enzymes (e.g., heme oxygenase-1, reductase, aldose), while glyceraldehyde-3-phosphate dehydrogenase activity is downregulated and extracellular signal-regulated kinases ERK-1 and ERK-2 are inactivated. These activities cause genomic damage and disrupt the cell cycle. DNA methylation is observed in the case of Cr and Cd exposure (Edwards and Myers, 2007; Arita and Costa, 2009). Trivalent Cr is essential in trace quantities for normal reproductive functions. While, it is chronic exposure leads to significant epigenetic alterations in sperm influencing parental imprinting, ovarian cyst formation, uterine anomalies, and tumors in male offspring. Trivalent As and bivalent Cd exposure affects cell associations in urothelial cells, lowering SPARC expression and playing a role in bladder tumor development (Larson et al., 2010). Prolonged Pb exposure increases superoxide dismutase (SOD) enzyme activity in neoplasia, suggesting a potential mechanism for reactive oxygen species production and carcinogenesis (Kasperczyk et al., 2004). These examples provide evidence that heavy metal exposures cause epigenetic alterations, linking heritable changes in gene expression to increased likelihood of various physiological disorders, including reproductive issues.

### Monitoring of toxic metals contamination

Toxic metals stemming from escalating human activities necessitate increased monitoring due to their accumulation in the environment and animal tissues. This monitoring is crucial for estimating health hazards for both humans and animals as it involves identifying environmental contaminants and assessing affected areas (Khan et al., 2018). Estimating metal contents in animal tissues aids in diagnosing environmental pollution, accomplished through various biological techniques including the use of bio-indicators (Khan et al., 2019). Animal exposure to toxic metal poisoning is primarily influenced by contaminated feeds containing toxins like pesticides and metals (Khan et al., 2023). Monitoring animal feeds for toxic metal contamination is imperative especially in areas with polluted soils or industrial wastes (Usman et al., 2022).

The accumulation of toxic metals in the soil which enters the food chain through plants poses a direct threat to humans consuming animal products (Năstăsescu et al., 2020). Despite the accumulation of toxic metals in animal tissues, a lack of international standards for certain elements in food products poses risks to consumer health (European Commission, 2006). Toxic metal bio-monitoring using blood plasma, urine, or hair samples offer insights into metal concentration and excretion patterns. Hg excretion occurs in urine and feces, while Pb is found in hair, blood, and urine samples (Hsieh et al., 2019). As can be detected in blood, feces, and keratin tissues hours after exposure. Diagnosis of toxic metal poisoning in cattle also involves testing milk, an indirect indicator of environmental pollution (Pšenková et al., 2020).

Toxic metals predominantly accumulate in internal organs making it feasible to establish standard levels in the body (Ke et al., 2023). Diagnosis and control of contamination in animal feed ensure the safety of human food and health. High concentrations of Cd and Hg in animal feed (>10 ppm) are considered toxic, while Co, Cu, Pb, Mo, or Ba are regarded as toxic at concentrations above 40 ppm (Mc Geehan et al., 2020). Mandatory bio-monitoring programs should be established, requiring regular testing of blood, body fluids and tissue samples from livestock is essential, as relying solely on feed testing is insufficient for detecting metal contamination (McGeehan et al., 2020). These programs, overseen by veterinary authorities or certified laboratories, should set safe thresholds for heavy metal concentrations. Additionally, state authorities must monitor and regulate industrial activities that contribute to metal contamination, implement education programs for livestock owners, and enforce penalties for non-compliance. Investing in research and fostering inter-agency collaboration will further strengthen efforts to prevent heavy metals toxicities in livestock environments.

Further, Collaborative efforts between veterinary, environmental, agricultural, and public health agencies are essential to address heavy metal contamination comprehensively. Public awareness campaigns should also be launched to educate farmers and the public about the risks of heavy metal contamination in livestock, while investments in testing facilities and laboratories will bolster monitoring efforts (Chen et al., 2022). Additionally, policy development and implementation, alongside surveillance systems to monitor contamination trends, are crucial for prompt response to outbreaks or incidents of toxicity. Through these measures, state authorities can safeguard livestock health and ensure the safety of the food supply chain.

### Prevention and control of bioaccumulation of toxic metals

Soil remediation is used to reinstate the effectiveness of soil that indirectly reduces the chances of toxic metal exposure and their harmful effects (Xu et al., 2021). The techniques used for remediation of soil are based on biological and/or chemical protocols (Mahey et al., 2020). Bioremediation is used to clear water and soil from toxic metals that are harmful for living beings. This process utilizes a variety of plants and different microorganisms for biological restoration of contaminated areas. Soil can be reclaimed from Cu contents by using *Pseudomonas aeruginosa* and *Bacillus* spp. whereas phytoremediation is helpful in the clearance of Co from soil and water. The toxic metals are immobilized in plants, thus their further spread is prevented. The symbiotic bacteria of these plants possess the potential to adsorb these toxic metal pollutants. Chemical techniques for reduction of toxic metal pollutants reduce the bioavailability of metals (Singh and Prasad, 2015).

Environmental contamination with toxic metals extends to animal feed, leading to potential risks upon consumption (Lopez-Alonso et al., 2017). Contaminated animal feed introduces metals into animal bodies, eventually excreted in urine and feces (Hao et al., 2019). Animal dung rich in proteins and minerals may act as a fertilizer but can also be a source of toxic metal contaminants (Li et al., 2020). Composting reduces metal concentrations in manure by altering physicochemical properties and utilizing microorganisms to immobilize and oxidize metals (Hao et al., 2019; Wang et al., 2019). Landfills contribute to environmental pollution with toxic metals, generating leachate that contaminates soil and groundwater. Phytoremediation, using plants to extract and accumulate metals, aids in the recovery of landfill soils. Biochar produced during biomass pyrolysis reduces the bioavailability of toxic metals, improving soil quality. The deposition of sewage sludge in landfills poses a contamination risk but pyrolysis transforms sewage sludge into biochar, reducing metal spread and increasing soil pH (Penido et al., 2019; Elbehiry et al., 2020). The use of biochar in sewage sludge treatment minimizes pollution risks and enhances soil fertility (Penido et al., 2019; Li et al., 2020).

## Conclusion

Heavy metal toxicity has emerged as a major environmental challenge with detrimental impacts on livestock health and productivity. Heavy metals accumulate in the environment and then infiltrate animals from both natural and anthropogenic sources. Despite the essential role of some metals in maintaining biochemical and physiological functions, all metals exert their toxic effects via metabolic interference and mutagenesis. These metals affect various organs in animals, with the liver being typically the first impacted followed by the kidneys, brain, and reproductive system. The accumulation of heavy metals results in both gross and histopathological changes, ultimately leading to impaired growth and production. Prevention is paramount in addressing heavy metal contamination in livestock. Phytoremediation and intercropping can help to remove heavy metals from the environment before ingestion by animals. If heavy metals do enter the body, entero-absorbents can be employed to chelate them. Additionally, heavy metals commonly induce oxidative stress through various mechanisms, leading to altered redox potential, so including antioxidants in the therapeutic regimen has shown promise in significantly reducing the toxic effects associated with heavy metal exposure. Moreover, upcoming research will benefit from evaluating innovative targets as protective measures against organ toxicity caused by heavy metals.
